# Retrospective longitudinal study on the long-term impact of COVID-19 infection on polysomnographic evaluation in patients with Prader-Willi syndrome

**DOI:** 10.1186/s13023-024-03447-9

**Published:** 2024-12-13

**Authors:** Sina Braun, Constanze Laemmer, Sandra Schulte, Bettina Gohlke

**Affiliations:** 1 Department of Paediatric Endocrinology and Diabetology, Children`s Hospital, University of Bonn, Venusberg Campus 1, Building 30, 53127 Bonn, Germany; 2 Paediatric Endocrinology, Dept. of Paediatrics and Adolescent Medicine, KJF Klinikum Josefinum, Joseph-Mayer-Straße 1, 86154 Augsburg, Germany; 3 Paediatric Endocrinology, St. Bernward Krankenhaus GmbH, Treibestraße 9, 31134 Hildesheim, Germany

**Keywords:** Prader-Willi syndrome, COVID-19, Long COVID, Polysomnography, COVID-19 sequelae

## Abstract

**Background:**

To evaluate the impact of coronavirus disease 2019 (COVID-19) on polysomnographic evaluation in patients with Prader-Willi syndrome (PWS).

**Patients and methods:**

A retrospective cohort study of two consecutive overnight polysomnograms (PSG) in 92 PWS patients (mean age 9.1, range 3.1–22 years). 57/92 participants (35 female) had a COVID-19 infection between the two consecutive examinations. 35 patients (21 female) had no infection (control group). Distribution of genetics was as follows: 13/57 (22.8%) deletion, 19/57 (33.3%) uniparental disomy, 2/57 (3,5%) imprinting defect, 3/57 (5.3%) non-deletion, 20/57 (35.1%) diagnosed by analyses of the methylation pattern of chromosome 15q11-13. Mean time interval between COVID-19 infection and post-COVID-19 evaluation was 96.2 days.

**Results:**

Course of COVID-19 infection was asymptomatic 8/82 (9.8%), mild 63/82 (76.8%), medium 11/84 (13.4%). The five most frequently experienced symptoms in PWS patients were fever (56.1%); headache (45.1%); cold (42.7%); cough (31.7%) and body aches (21.95%). PWS patients who had COVID-19 infection had significantly lower mean oxygen saturation (SpO2) measured by pulse oximetry (post 94.8% vs. pre 95.7%, *p* = 0.001), lower detected lowermost SpO2 (post 86.2 vs. pre 87.3%, *p* = 0.003), and higher occurrence of hypopnoea (post 13.9 vs. pre 10.7, *p* = 0.001). Time in optimal SpO2 (95–100%) decreased significantly (post 54.3% vs. pre 73.8%, *p* = 0.001), whereas an increase was observed in time in suboptimal SpO2 (90–95%) (post 45.5% vs. 25.8%, *p* = 0.001) and in time in poor SpO2 (< 90%) (post 0.7% vs. pre 0.2%, *p* = 0.030). Body-Mass-Index (BMI)-SDS for PWS showed no differences between the groups at any time. BMI-SDS-differences showed no influence on differences in SpO2 evaluations. In the genetic subgroup with deletion there was a statistically significant effect on an increased number of OSA (*p* = 0.027). The genetic subgroup with uniparental disomy (UPD) was associated with a reduced risk of higher HF (*p* = 0.035) and less hypopnea (*p* = 0.011).

**Conclusion:**

PWS patients predominantly experienced only mild to medium symptoms during COVID-19 infection without necessity of hospitalisation. However, on average three months after infection, differences in PSG evaluations were still apparent, manifesting in lower SpO2 and more frequent hypopnea. A long-lasting impairment of the pulmonary system due to the COVID-19 infection might be responsible.

## Introduction

Prader-Willi syndrome (PWS) is a genetic disorder affecting 1/10,000 to 1/20,000 live births. It is caused by changes on segment 15q11-q13 of the paternal chromosome and attributed to paternal deletion (60%), maternal uniparental disomy (35%) or imprinting defects [1]. Clinical symptoms change with age, starting with central hypotonia, reduced muscle mass, and feeding problems after birth, which can cause respiratory distress and asphyxia [2]. In early childhood, weight gain occurs around two years of age followed by increased interest in food and then hyperphagia by age five. This leads to weight gain and obesity if energy intake is not controlled [1–3]. The PWS phenotype is characterized by short stature with small hands and feet due to hypothalamic dysfunction [4, 5] and growth hormone treatment [2] is recommended as soon as the diagnosis is made and after adequate nutrition has been achieved, usually between three to four month of age [1, 6].

Because of a high prevalence of sleep disorders and respiratory dysfunction, including oxygen desaturation, central hypoventilation, and obstructive sleep apnoea (OSA), overnight polysomnography (PSG) and the monitoring of oxygen saturation measured by pulse oximetry (SpO2) is recommended annually from childhood onwards [1, 2, 7, 8].

As a reaction to a rapid worldwide spread of novel coronavirus disease 2019 (COVID-19), the World Health Organization declared a global pandemic in March 2020 [9]. COVID-19 clinically manifests itself variably with cough, fever, sore throat, anosmia, and respiratory problems such as dyspnoea [10, 11]. Children are more likely to have mild symptoms or remain asymptomatic during throughout infection [11]. Risk factors for a higher symptom burden are age above 60 years, obesity, male sex, and comorbidities such as OSA, diabetes, hypertension, cardiovascular, and chronic kidney diseases [12, 13]. In paediatric patients obesity is particularly associated with more severe outcomes of a COVID-19 infection [12, 14]. Although patients with PWS seem to be an at-risk group for COVID-19 infection, two prior studies on children and adolescents with PWS showed only a low severity of infection [15, 16]. These studies collected data on the acute course of infection using questionnaires, but did not measure long-term consequences using medical parameters. To our knowledge, there are no studies about possible long-term sequalae of a COVID-19 infection in PWS patients. The aim of this study was to collect data on the acute course of infection as well as on possible long-term sequalae. We studied overnight PSG examinations in PWS patients before and after COVID-19 infection as well as data on the acute course of infection using a questionnaire.

## Methods

### Study design and setting

In this retrospective and longitudinal study, we collected auxologic and PSG data on 92 children, adolescents, and young adults with PWS who visited a German PWS center for follow-up treatment between April 2022 and November 2022. All members of our cohort had previously participated in the study on effects of COVID-19 lockdown by Mohr et al. in 2021 [17].

**Fig. 1:**
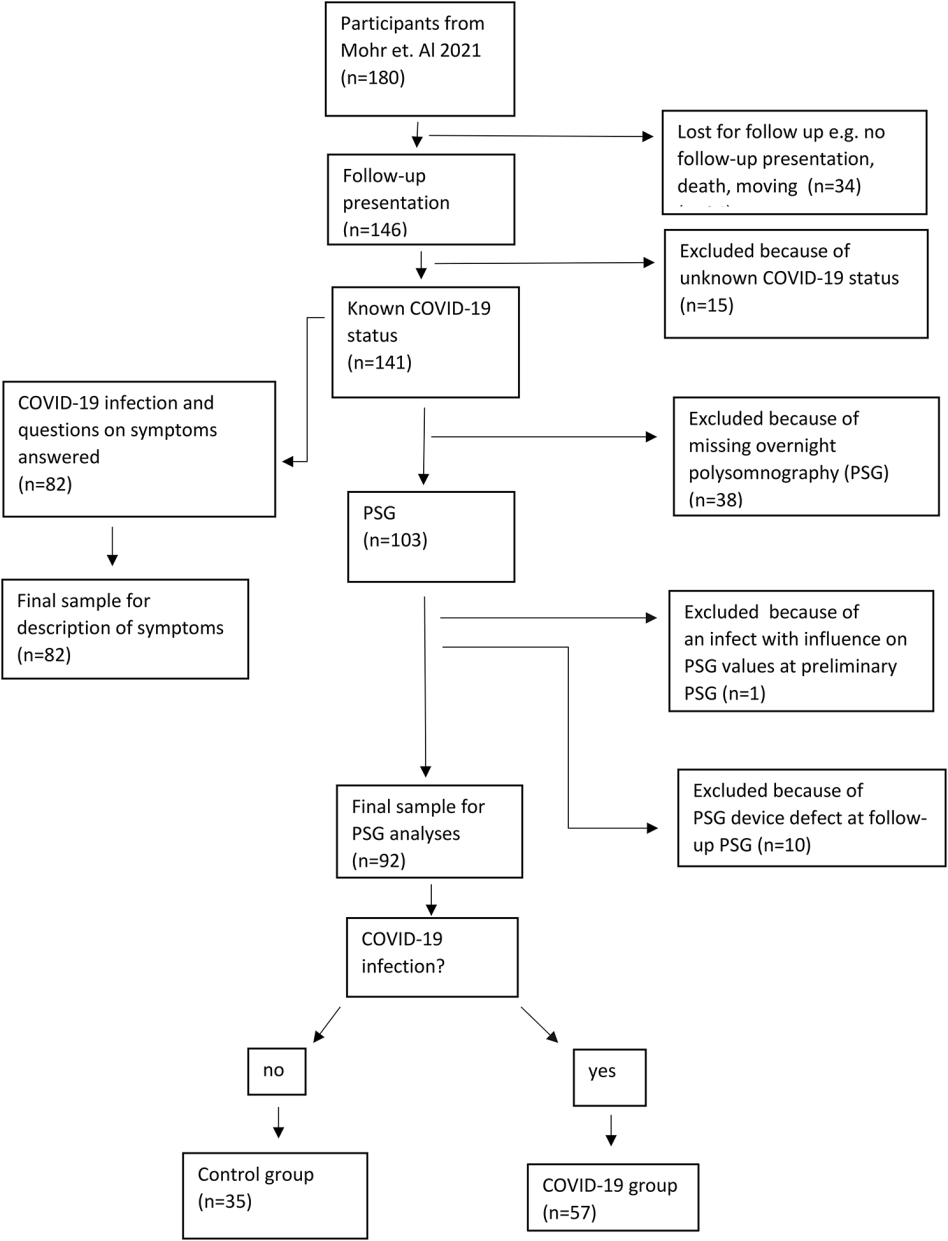
Flow chart of included and excluded patients, COVID-19 and Control group

Inclusion criteria were two valid PSG examinations and information about the COVID-19 infection status. For detail see Fig. 1 (flow chart). There were 57/92 (64%) participants (35 female) who contracted COVID-19 infection in the time between the first and second PSG. The control group who did not contract COVID-19 consisted of 35 participants (21 female). We compared PSG data from before and after a COVID-19 infection and compared it to the latest two examinations at the PWS center during the same time interval from the control group. Mean time difference between the preliminary and follow-up examination was 6.93 months, 6.85 for the group with the COVID-19 infection and 7.05 for the control group.

Mean age was 9.1 with a range from 3.1 to 22 years. There were 78/92 (84.8%) participants treated with growth hormone. The genetic subtype was determined in 72/92 (78.3%) of which 33/72 (45.8%) had a deletion, 28/72 (38.9%) a uniparental disomy, 6/72 (8.3%) an imprinting defect, and 5/72 (6.9%) a non-deletion (not differentiated into uniparental disomy or imprinting defect). In 20/92 (21.7%), the genetic subtype was unknown, as PWS was diagnosed by an analysis of the methylation pattern of chromosome 15q11-13. In the COVID-19 group, the distribution of genetics was as follows: 13/57 (22.8%) deletion, 19/57 (33.3%) uniparental disomy, 2/57 (3,5%) imprinting defect, 3/57 (5.3%) non-deletion, 20/57 (35.1%) diagnosed by analyses of the methylation pattern of chromosome 15q11-13.

Further, in the COVID-19 group, 54/57 (94.7%) of parents or guardians gave information on the COVID-19 vaccination status of their children before their COVID-19 infection. There were 32/54 (59.3%) who had not been vaccinated, five (9.3%) once, twelve (22.2%) twice and five (9.3%) three times before their COVID-19 infection. In the control group, 19/35 (54.3%) had not been vaccinated, nine (25.7%) twice (25.7%), six (17.1%) three and one (2.9%) four times against COVID-19.

None of the participants received prophylactic steroid treatment for adrenal insufficiency, and none of the participants received treatment with continuous positive air pressure (CPAP) pre, during or post COVID-19 examination.

Additionally, in 30 participants who had contracted COVID-19 infection, PSG was not available. These participants had to be excluded from the final sample (*n* = 30), but we have taken their questionnaires into account concerning evaluation of symptoms (see Fig. 1, flow chart).

All procedures performed in this study involving human participants were in accordance with the ethical standards of the University Hospital Bonn and with the 1964 Helsinki Declaration and its later amendments. Ethical approval was given by the institutional Ethics Committee of the University of Bonn, Germany (ethics committee number: 31/15; June 24, 2020). All parents or guardians gave written informed consent for participation.

### Respiratory analysis

For respiratory analysis and SpO2 measurements, a SOMNOtouch™ RESP by SOMNOmedics was used as a PSG device, including paediatric sensors for children. Basal SpO2, lowermost SpO2, number of desaturations, oxygen desaturation index (ODI), amount of sleep time in optimal saturation range (95–100%), suboptimal saturation range (90–95%), and poor saturation range (85–90%) were measured. If a nasal cannula was tolerated, hypopnea, OSA, mixed apnoea, and central apnoea were differentiated. An electrocardiogram measured heart rate.

### Body composition

Height was measured using a standardized rigid stadiometer and weight was measured using a standardized mechanical scale for weight measurement to the nearest 0.1 kg. Body Mass Index (BMI) was calculated as weight/height^2^ and standard deviation scores (SDS) adapted to age and sex for height, weight, and BMI were calculated. Reference values for children with PWS (SDS_PWS_) [18] and healthy German children (SD _healthy_) [19] were used.

### The questionnaire

**Fig. 2:**
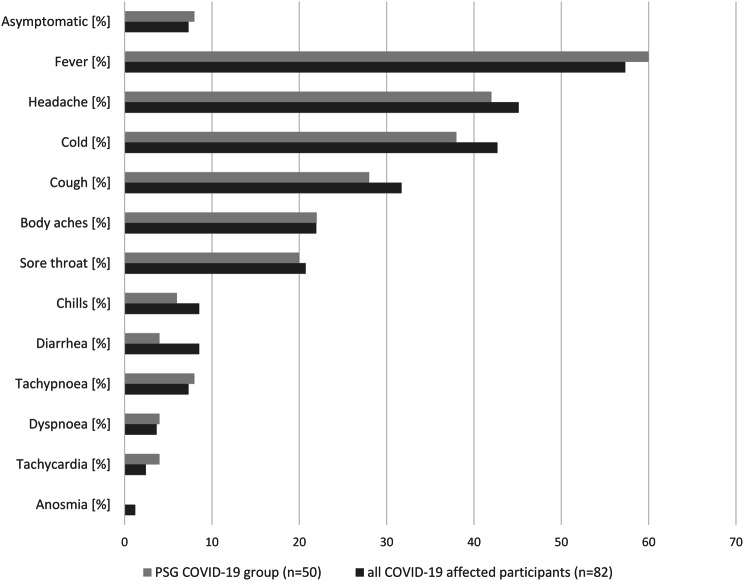
Reported symptoms of COVID-19 infection in PWS patients

The participants’ parents or guardians answered our questionnaire retrospectively between November 2022 and January 2023 via e-mail or telephone. The amount of time that had passed since the COVID-19 infection therefore varies accordingly. All COVID-19 infections occurred between January 2020 and November 2022, with only one participant having an infection in 2020, six in 2021, and the majority of our participants having been infected with COVID-19 in 2022. Questions on COVID-19 infections included date of diagnosis, onset of symptoms, type of testing (personal antigen-test, official antigen-test from a certified test centre or PCR) and symptoms experienced. In asymptomatic participants the positive test result was detected by routine tests at school, the residential facility or when taking part in support measures and leisure activities. The symptoms we included into the questionnaire were inspired by German COVID-19 report DEMIS (Robert Koch Institute). We subdivided the COVID-19 group into a mild, medium, and severe COVID-19 infection based on the reported symptoms by the parents or guardians and long-term impact of the COVID-19 infection (see Fig. 2). Low severity was defined as an asymptomatic infection or an infection including symptoms two to ten of Fig. 2 during the COVID-19 infection (see Fig. 2). Medium severity was defined as incomplete remission of all symptoms after infection as reported by caregivers, secondary disease, dyspnoea, tachypnoea, or tachycardia. A severe COVID-19 infection was defined as requiring ventilation, intensive care unit treatment, or death. The parents or guardians were asked to give information on COVID-19 vaccination status and how the vaccination was tolerated by their children. Additionally, data on other infections besides COVID-19 within the last year were collected.

### Statistical analyses

Statistical analyses were carried out with IBM SPSS Statistics (version 27). Standard deviation scores were calculated for height, weight, and BMI, based on German reference values [19] and PWS reference values [18]. If patients were older than 18 years, SDS values for 18 years were used. Clinical data and results of PSG are reported as means and standard deviations (SD). Delta values (Δs) indicate differences between pre and post COVID-19 follow-up. To calculate Δ, for the BMI-SDS difference, the data from the pre-infection-exam were always subtracted from the data of the post COVID-19 follow-up (post COVID-19 BMI – pre COVID-19 BMI = individual Δs). No significant differences in post-pre-differences (Δs) were found between female and male pairs, making a further division into female and male subgroups superfluous. Before testing, each parameter was always evaluated for normal distribution. If the assumption of normality did not hold or group size was *n* < 10, non-parametric testing was performed. For measurements on ratio scales e.g., auxological data and results of PSG, a paired t-test or the Wilcoxon signed-rank test were used to compare pre- and post-values. For analyses between COVID-19 and control group, independent sample t-test or Mann-Whitney-U-Test were used. In order to further analyse determinants of differences in sleep laboratory examinations, we performed stepwise multiple linear regression analyses.

The individual Δs were always used as dependent variables. Independent variables included.

into the analysis were: sex, age, and ΔBMI-SDS_healthy_. To further analyse the influence of COVID-19 infection, a second model of stepwise multiple linear regression analyses was performed, with COVID-19 symptoms (ordinal, 0 = no symptoms, 1 = mild symptoms, 2 = moderate to severe symptoms) and time since COVID-19 infection in days as independent variables. We decided to perform two separate analyses instead of one approach to avoid overfitting of the model with *n* = 57 in the COVID-19 group, as advised by Babyak and Rothman (p. 226 of 2,3). To further analyse if the individual Δ (= post COVID-19 – pre COVID-19) was influenced by the genetic subtype, dummy variables were coded for each genetic subtype. Stepwise linear regression was performed for each individual Δ of all parameters with all of the dichotomous variables for genetic subtypes. Cohen’s f² values were calculated to estimate effect sizes. Significance was defined as *p* ≤ 0.05, a trend as *p* > 0.05 < 0.1. Since this work represents an exploratory data analysis, no adjustments for multiple testing were performed [20, 21].

## Results

### Severity of illness and symptoms of COVID-19 infection

Figure 2 shows the reported symptoms of COVID-19 infection for all affected patients and for those in which repeated PSG was available.

According to the reported symptoms we classified the course of the COVID-19 infection as asymptomatic in 8/82 (9.8%), mild in 63/82 (76.8%), and medium in 11/82 (13.4%). There were no severe outcomes in our study cohort. Fatigue (*n* = 6, 7.3%), exhaustion (*n* = 5, 6.1%), stomach ache (*n* = 2, 2.4%), night sweats (*n* = 2, 2.4%), hair loss (*n* = 1, 1.2%) and loss of appetite (*n* = 1, 1.2%) were reported as free text answers in the question on other symptoms. No parents or guardians reported a loss of taste or pneumonia. None of our participants was hospitalised. Only one participant visited the emergency room, because of shortness of breath and received outpatient treatment.

In 78/82 (95.1%) there was a total remission of all symptoms reported by caregivers. Three caregivers claimed that their child is still less resilient than before the infection and one participant developed rheumatism of the ankle and hand joint.

### Inter-group differences at pre-examination

There were no statistically significant differences for PSG measured between the COVID-19 and control group at the pre-COVID examination apart from more frequent mixed apnoea in the COVID-19 group (COVID-19 group 2.56 vs. control group 1.00; *p* = 0.042). Table 1 illustrates this in more detail (see Table 1).

**Table 1:** Intra-group comparison between pre/post data in the control group; intra-group comparison pre/post COVID-19 infection in the COVID-19 group and inter- group comparison (COVID-19 vs. Control group) at pre and post examination

	8.3 years; 3.1–22.0					9.5 years; 3.2–20.9					
	Intragroup pre vs. post examination (Control group) *n* = 35					Intragroup pre vs. post COVID-19 (COVID-19 group) *N* = 57					Intergroup Control vs. COVID-19 group (*n* = 92)
	n	Result control group	SD	p	ranks - ; + ; =	n	Result COVID-19 group	SD	p	ranks - ; + ; =	p
HF post [bpm]	35	88.20	18.61	0.028	22; 12; 1	57	85.44	13.55	ns	29; 25; 3	ns
HF pre [bpm]	35	92.14	18.42			57	86.72	16.18			ns
BMI post [kg/m^2^]	35	17.37	3.32	0.006	10; 25; 0	57	19.34	5.42	0.003	16; 37; 3	0.086
BMI pre [kg/m2]	35	17.01	3.20			57	18.78	5.02			ns
BMI SDS_healthy_^1^ post	35	0.15	1.03	ns	12; 23; 0	57	0.56	1.23	0.014	24; 32; 1	ns
BMI SDS_healthy_^1^ pre	35	0.03	1.04			57	0.40	1.25			ns
BMI SDS_PWS_^2^ post	35	-1.41	0.50	ns	19; 15; 1	57	-1.29	0.73	ns	29; 28; 0	ns
BMI SDS_PWS_^2^ pre	35	-1.40	0.65			57	-1.29	0.77			ns
mean SpO2 post [%]	35	95.31	1.68	0.083	20; 11; 4	57	94.82	1.54	0.001	30; 10; 17	ns
mean SpO2 pre [%]	35	95.86	1.56			57	95.65	1.40			ns
lowest SpO2 post [%]	35	87.89	3.72	ns	17; 9; 9	56	86.21	5.16	0.003	30; 12; 14	ns
lowest SpO2 pre [%]	35	88.83	2.94			56	87.29	10.79			ns
desaturations post [no.]	35	21.94	13.46	ns	18; 16; 1	54	26.65	20.37	ns	20; 31; 3	ns
desaturations pre [no.]	35	24.83	22.75			54	29.78	34.42			ns
ODI post	33	2.86	1.80	ns	17; 14; 2	52	3.44	2.45	ns	23; 26; 3	ns
ODI pre	33	3.31	3.09			52	3.83	4.02			ns
OSA post [no.]	26	12.23	9.62	ns	12; 13; 1	38	17.5	13.25	ns	15; 22; 1	0.095
OSA pre [no.]	26	14.62	17.35			38	16.53	13.87			ns
hypopnoea post [no.]	22	9.55	6.97	ns	10; 12; 0	35	13.91	13.38	0.035	11; 22; 2	ns
hypopnoea pre [no.]	22	7.68	6.12			35	10.66	9.56			ns
CA post [no.]	10	5.40	3.10	ns	2; 8; 0		6.06	4.35	ns	10; 6; 1	ns
CA pre [no.]	10	4.30	3.68			17	6.29	5.27			ns
MA post [no.]	4	2.00	0.82	ns	0; 3; 1	9	2.00	1.32	ns	3; 2; 4	ns
MA pre [no.]	4	1.00	0.00			9	2.56	1.88			0.042
time 95–100 SpO2 post [%]	35	68.97	34.76	ns	21; 13; 1	55	54.29	36.85	0.001	39; 13; 3	0.074
time 95–100 SpO2 pre [%]	35	78.29	29.30			55	73.79	28.49			ns
time 90–95% SpO2 post [%]	35	29.53	34.99	ns	14; 20; 1	55	45.49	36.78	0.001	13; 39; 3	0.042
Time 90–95% SpO2 pre [%]	35	20.87	28.88			55	25.81	28.23			ns
time < 90% SpO2 post [%]	35	0.27	0.71	ns	6; 7; 22	55	0.70	2.66	0.030	4; 16; 35	
Time < 90% SpO2 pre [%]	35	0.18	0.59			55	0.22	0.80			ns

^1^=Standard deviation scores for healthy children, ^2^=Standard deviation scores for children with Prader-Willi syndrome, ranks: - indicates post < pre, + indicates post > pre, = indicates post = pre

Abbreviations: HF = Heart rate, BMI = Body Mass Index, SpO2 = Oxygen saturation measured by pulse oximetry, no. = Number of, ODI = Oxygen desaturation index, OSA = Obstructive sleep apnoea, CA = Central sleep apnoea, MA = Mixed sleep apnoea, time 95–100%= Time in optimal saturation range, time 90–95%= Time in suboptimal saturation range, time < 90%= Time in poor saturation range

### Intra-group differences between two consecutive examinations (control group) or pre and post COVID-19 infection (COVID-19 group)

**Fig. 3:**
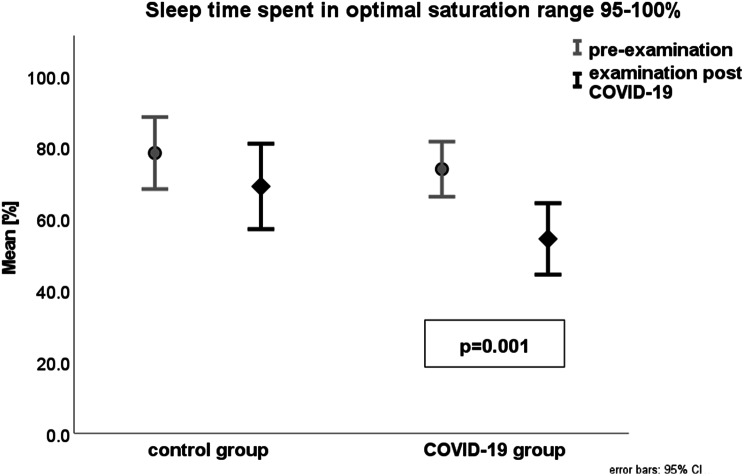
Sleep time spent in optimal saturation range (SpO2 95–100%) pre (grey) and post (black) COVID-19 infection in 57 PWS patients

**Fig. 4:**
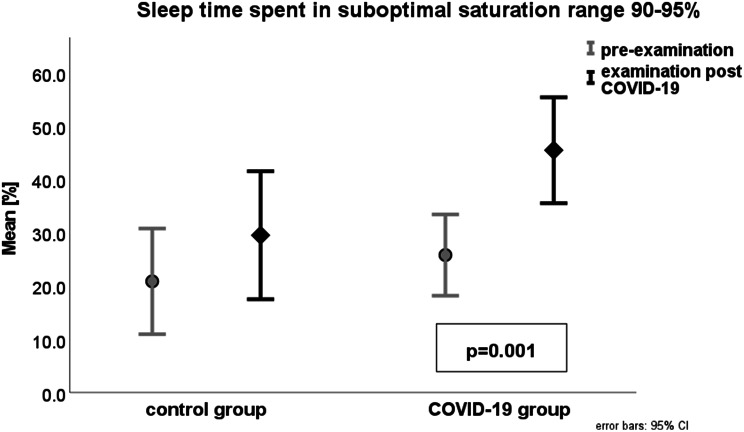
Sleep time in reduced saturation range (SpO2 90–95%) pre (grey) and post (black) COVID-19 infection in 57 PWS patients

In the COVID-19 group, we found a statistically significant reduction of sleep time spent in optimal saturation range of 95–100% (post 54.3% vs. pre 73.8%; *p* = 0.001) (Fig. 3) and an increase in time in 90–95% saturation range (post 45.5% vs. pre 25.8%; *p* = 0.001) (Fig. 4), For all data, see Table 1. The average time interval between infection and post-COVID-19 evaluation was 96.2 days, with a range from five to 402 days.

**Table 2**: Stepwise multiple linear regression analyses of Δ sleep laboratory (Δ = post Covid – pre Covid), ^1^ 0 = female, 1 = male, ² 0 = without symptoms, 1 = mild symptoms, 2 = moderate/severe symptoms. Abbreviations: SpO2 = oxygen saturation measured by pulse oximetry, ODI = oxygen desaturation index, OSA = obstructive sleep apnoea, CA = central sleep apnoea, MA = mixed sleep apnoea, HF = heart rate.

### Stepwise multiple regression analyses of the COVID-19 subgroup

To analyse factors influencing the change in the COVID-19 group between post COVID-19 and pre COVID-19 examination, we performed three multiple regression analyses of Δ = post-COVID-19 – pre COVID-19 (Table 2).

In the first multiple regression (results with influence see Table 2), age, gender and ΔBMI Kromeyer showed no influence on Δ: mean SpO2, lowest SpO2, time spent in optimal saturation range (95–100% SpO2), time spent in suboptimal saturation range (90–95% SpO2), time spent in poor saturation range (< 90% SpO2), number of hypopnoeas, CA, and MA.

**Table 2:** Stepwise multiple linear regression analyses of Δ sleep laboratory (Δ = post COVID-19 – pre COVID-19) ^1^0 = female, 1 = male

	Stepwise multiple linear regression analyses in COVID-19 subgroup			
	Independent variables regression standardised coefficients (ß)			
Dep. variables	Sex^1^	Age	Δ BMI SDS_healthy_	*R*^2^ corr.
Δ desaturations	*p* = 0.086	0.303 *p* = 0.026		0.075, p = **0.026** F = 5.27, f²=0.081
Δ ODI	*p* = 0.063	0.280 *p* = 0.045		0.060, *p* = 0.045 F = 4.24, f²=0.064
Δ OSA	-0.353 *p* = 0.030			0.100, *p* = 0.030 F = 5.12, f²=0.11

**Abbreviations**: BMI SDS_healthy_= body mass index standard deviation score for healthy German children, ODI = oxygen desaturation index, OSA = obstructive sleep apnoea, HF = Heart rate

In a second regression, days passed between COVID-19 infection and PSG evaluation post-COVID-19 infection and symptom severity showed no influence on any of the Δ respiratory parameters analysed.

**Fig. 5:**
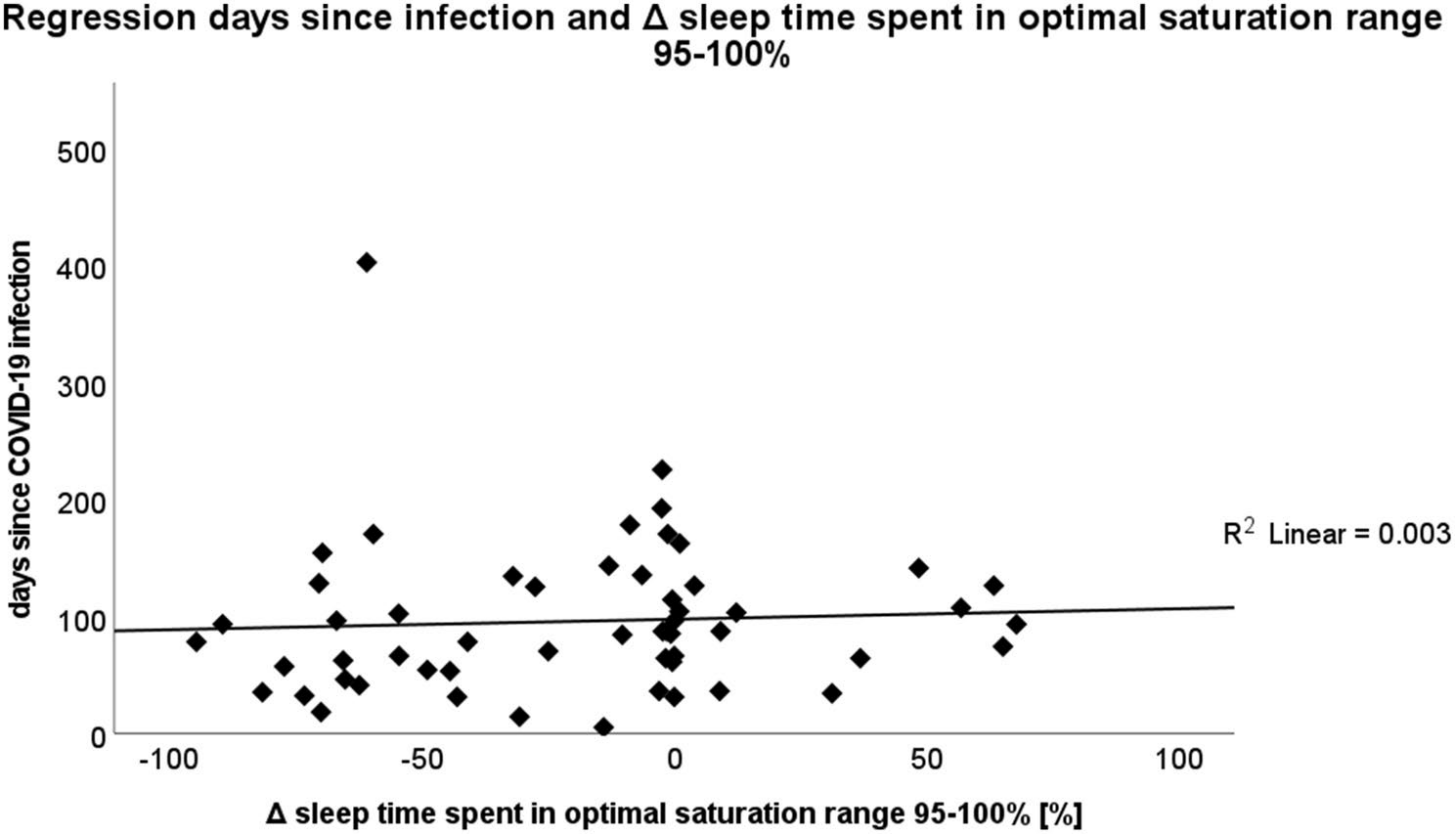
Regression days since infection and Δ sleep time spent in optimal saturation range 95–100%

There was no influence of days passed between COVID-19 infection and PSG evaluation post-COVID-19 infection on the Δ of sleep time spent in optimal saturation range of 95–100% (Fig. 5).

A third multiple regression analysis showed different effects on Δ HF, ΔOSA and Δ hypopnea in the different genetic subgroups (see Table 3).

**Table 3:** Stepwise multiple linear regression analyses of Δ sleep laboratory (Δ = post COVID-19 – pre COVID-19)

	Stepwise multiple linear regression analyses in COVID-19 subgroup		
	Independent variables regression standardised coefficients (ß)		
Dep. variables	Deletion	UPD	*R*^2^ corr.
Δ HF		-0.280 *p* = 0.035	0.062, *p* = 0.035 F = 4.7, *n* = 57, f²=0.07
Δ OSA	0.350 *p* = 0.027		0.105, *p* = 0.027, F = 5.33, *n* = 38, f²=0.12
Δ hypopnoeas		-0.425 *p* = 0.011	0.156, *p* = 0.011, F = 7.27, *n* = 35, f²=0.18

Abbreviations: ODI = oxygen desaturation index, OSA = obstructive sleep apnoea, HF = Heart rate, UPD = uniparental disomy

## Discussion

With this study on the acute course of COVID-19 infections in PWS patients and the long-term impact on polysomnographic evaluation, we found subtle but significant and lasting changes detected by PSG. It was hypothesized that patients with PWS would be an at-risk group for a severe course of a COVID-19 infection because of the syndrome associated with obesity and respiratory dysfunction. However, in accordance with others [15, 16], we found only mild to moderate impairment directly associated with the infection. However, through analysis of PSG parameters and oxygen saturation, long-term lasting changes to the respiratory system were detected.

Similar to the two mentioned studies [15, 16], respiratory problems and breathing difficulties were only reported twice, tachypnoea occurred four times in our cohort. Fever was the most frequently observed symptom reported by caregivers in our study cohort. Despite the potential for lack of appropriate febrile response, which is often seen in PWS patients due to disturbed thermoregulation [22], we suggest that fever or raised body temperature is a reliable symptom of COVID-19 infection in PWS patients, as it is in healthy individuals [10, 23]. In general, in our study the reported clinical symptoms during the infection observed by caregivers did not differ from those already reported in studies on healthy children [10, 23] and is in accordance with other studies including children and adults with PWS [15, 16]. Thus, we assume that PWS patients have similar symptom profiles in comparison to the general population during the acute infection.

Although the severity of infection was only mild to moderate, and the caregivers reported a total remission of the symptoms in almost all participants, there were statistically significant differences in PSG at follow-up examination after an average of three months post-COVID-19 infection. Time of sleep spent in the target area of 95 to 100% oxygen saturation was significantly less in comparison to the examination before COVID-19 infection. The changes in the lowermost SpO2 and mean SpO2 were small, so the clinical relevance of these two changes remains unclear. However, this was clearly a directional trend since the majority of participants showed worsened lowest and mean SpO2 after COVID-19 infection. Moreover, there was a statistically significant shift to more frequent hypopnoea in the post-COVID-19 follow-up examination. This is in accordance with what has already been reported in non-PWS COVID-19 patients. Low oxygen saturation without dyspnoea during the infection and during the acute rehabilitation phase has been described as “happy or silent hypoxia” [24–30]. The reason for the discrepancy between decreased perception of oxygen deficiency and the absence of the feeling of dyspnoea is not fully understood [30]. However, reduced SpO2 during the COVID-19 infection seems to affect PWS patients, as it has been described in a previous study on children and adults with PWS [16]. Whittington et al. reported that “low measured oxygen saturation” was mentioned as a symptom, although it was not explicitly asked for in their questionnaire. They reported that it occurred in “less than 10%” of the patients [16, 31]. It remains unclear, whether reduced SpO2 would have been mentioned more frequently, if it had been studied in routine SpO2 measurements in the acute phase, or if low measured SpO2 would have been added in the dropdown menu of the survey. SpO2 was not measured during the acute infection in our patients, because no patients were hospitalisedduring their infection.

PWS patients might be an at-risk group for “happy hypoxia” in COVID-19 infection. In earlier studies, Arens R and Gozal D et al. already showed a non-existent or significantly attenuated hypoxic ventilatory response in patients with PWS that was independent of the degree of obesity [32]. In a further study, they also showed a blunted hypoxic arousal response during sleep in PWS patients compared to a non-PWS control group [33]. They concluded that the primary abnormality of ventilatory control in PWS patients involves the peripheral chemoreceptor pathways [31–33]. Thus, an abnormal ventilation control with insensitivity to O2 and CO2 levels might contribute to low measured SpO2 with absence of dyspnoea in PWS patients with COVID-19 infection.

However, because there is still a reduction of SpO2 during sleep after an average of three months’ time after the acute COVID-19 infection, we assume that reduced SpO2 during sleep might be a persisting problem since the acute infection. Further studies are required to prove this hypothesis.

Raised BMI and obesity are strong confounders for sleep related breathing disorders (SRBDs) in simply obese children and children with obesity and PWS [34]. Therefore, we wanted to rule out that an increase in BMI might be the reason for the deterioration of SpO2 and more frequent hypopnea during sleep after an average of three months after COVID-19 infection. Mean BMI-SDS_PWS_ remained in the normal range, and the majority of our PWS patients were not obese. Additionally, BMI-SDS for healthy children remained in the normal range, despite a small but significant increase after COVID-19. However, multiple regression analysis of BMI SDS_healthy_ did not show statistically significant influence on the results of the respiratory SpO2 parameters. Thus, a change in BMI SDS_healthy_ could be due to PWS specific phenotypic and metabolic characteristics, as well as changes in nutritional and eating behaviour with increasing age [3, 22]. As these are not typical in children and adolescents without PWS, PWS syndrome specific BMI SDS are calculated [18].

Another possible explanation for our results might be alterations in pulmonary tissue or muscles of respiration. A study on adult non-PWS patients with OSA reported a significant increase in median CPAP pressure in their auto-CPAP treatment after COVID-19 infection [35]. Apnoea-hypopnoea index was slightly increased, but not significantly [35]. So, assuming there was no increased obstruction rate due to possible upper airway changes, e.g. persisting tonsil hyperplasia, this might imply some form of alteration in respiratory mechanics. We found no increase in obstructive sleep apnoea in our PWS patients after COVID-19 infection, but there was an increase in the number of hypopnoeas. More frequent shallower breathing due to alterations in respiratory mechanics might lead to a reduction of SpO2 measurement. This could be caused by changes in pulmonary tissue or muscles of respiration, or both.

To date, there have only been few studies in other paediatric patient populations comparing respiratory parameters in the same patients before and after COVID-19 infection and then comparing them with a non-COVID-19 control group. Mogensen et al. compared spirometric lung function tests in a population-based sample of young, healthy adults with and without asthma and a mild-to-moderate COVID-19 disease before COVID-19 infection with spirometric lung function tests after COVID-19 infection [36]. They found no evidence that an infection with COVID-19 resulted in impaired spirometric lung function [36]. In contrast, in a study by Soyak Aytekin et al. mean expiratory flow 25–75% values were significantly reduced after COVID-19 infection in some paediatric patients with asthma compared to prior lung function tests [37]. Thus, the low SpO2 values during sleep in our cohort might also be caused by a small airway dysfunction after COVID-19 [37].

Furthermore, a disturbed gas exchange after COVID-19 has already been discussed as a possible consequence of COVID-19 infection. Wu et al. reported in their study on 3-, 6-, 9-, and 12 months respiratory outcomes in adults without PWS who were hospitalized with severe COVID-19 infection, that diffusion capacity of the lungs for carbon monoxide was 77% of predicted at three months, 76% of predicted at six months, and increased to 88% of predicted at twelve months after hospital discharge [25]. The forced vital capacity also increased from 92% of predicted capacity after three months to 98% of predicted after twelve months [25]. As the diffusion capacity of the lungs for carbon monoxide improved with time [25], the question arises, whether the reduction in SpO2 during sleep in our study might be a consequence of a disturbed gas exchange and therefore, an improvement in SpO2 could also be expected with time. So far, our data does not indicate an improvement with time, as time since infection did not show an influence on PSG parameters in multiple regression analysis. As our study results are based on an average time span of three months after COVID-19 infection, further long-term studies are needed to better assess the time frame in which lowered SpO2 at sleep persists and to evaluate the potential resulting clinical implications of persisting increased sleep time in the lowered SpO2 range of 90–95% SpO2 after COVID-19 infection. For example, in a study by Lau et al.., an impact on verbal memory performance was associated with SpO2 nadir during sleep in children with OSA [38]. In their study, the group without OSA and better verbal memory performance showed a mean SpO2 nadir of 94.82% (range: 89–98) vs. 91.52 (range 76–97) in the OSA group with lower performance. The mean nadir in both groups was above 90%, once 92% and once 95%. So, it could be assumed that not only mean SpO2 < 90% might have clinical consequences, but also differences in the range of 90–100% with relatively more sleep time spent in lower intervals of 90–100%. Another possible clinical implication of reduced SpO2 during sleep is potential neurocognitive deficits, which are associated with sleep disordered breathing in children with sleep related breathing disorders [39]. Further studies are clearly needed to gain more data on clinical implications. Regular follow-up examinations by means of neurocognitive testing in combination with more frequent PSG are important to identify clinical implications after COVID-19 infection and should be considered for the regular care after COVID-19 infection.

Since cognitive disability and speech and language delay are known clinical manifestations in PWS [22], particular attention should be paid to the development of these parameters after COVID-19 infection, and possible treatment options should be discussed in time. CPAP, which is already used for PWS patients in the setting of OSA [40], could be considered as a possible treatment option in the case of persisting SpO2 below the optimal range during sleep after COVID-19 infection in PWS patients. According to a study by Turner et al., CPAP treatment had significant positive effects on working memory, long-term verbal memory, and short-term visuospatial memory in patients with OSA [41].

One limitation to our study is the possibility that some members of our control group did in fact contract COVID-19 during this time but remained asymptomatic and as such were not tested. Because of the retrospective study design, some symptoms the PWS patients experienced during their infection might not have been mentioned by the caregivers because of recall bias or because they have not been observed, due to a high pain threshold, which is a typical feature in people with PWS [1, 22].

The young age of our study cohort may have also contributed to a milder course of the disease [42–44].

Because of the low proportion of PWS patients with three or more vaccinations before their COVID-19 infection, no statistical analyses investigating the influence of vaccination status on course of COVID-19 infection and PSG parameters were performed.

Since we do not have a non-PWS control group, we cannot comment on whether the effect of COVID-19 infection on PSG parameters noted in this study apply to the general population. Whether a transfer of our results is possible should be investigated in future research. Moreover, we found only mild differences and the clinical implications remain unclear. However, all of our results are unidirectional and still apparent after approximately three months after COVID-19 infection, even if the majority of parents reported no persistent clinical overt symptoms. Further studies are needed to determine clinical significance and duration of the deterioration of PSG in patients with PWS.

The comparison between COVID-19 and the control group at post-examination showed a tendency towards more OSA in the COVID-19 group compared to the control group, but when analysing the COVID-19 subgroup, there were no statistically significant changes in HF and the number of OSA and hypopnoeas between pre- and post- COVID-19 examination. However, statistically significant differences were found when the genetic groups in the COVID-19 group were analysed separately. We observed that the deletion genetic subgroup had a statistically significant effect on an increased number of OSA, whereas the UPD genetic subgroup was associated with a reduced risk of higher HF and fewer hypopnoeas. There only have been few studies on the influence of genetics on cardiovascular and pulmonary risk in PWS patients so far. In an earlier study by Torrado et al.., more frequently present sleep disturbances were described in PWS patients with a deletion [45].

Moreover, a recent study by Cintra, Rocha et al. showed more frequent OSA in the deletion genetic group compared to the UPD group and 0% of the patients with an UDP had hypoventilation [46]. This is in accordance with the findings in our study and suggests that the genetic deletion may have a higher risk over time for sleep-disordered breathing and especially for OSA, whereas UPD might be associated with better progression over time. Future studies, with focus on the effect of genetics on cardiopulmonary parameters in a larger study cohort are of importance to prove this hypothesis.

The strengths of our study include the large patient population for PWS with COVID-19 infection, the large patient population without COVID-19 infection, and the assessment of longitudinal data. This allows us not only to compare changes pre- and post- COVID-19 for each participant individually, but also to compare the collectives, with and without COVID-19 infection. By regular follow-up examinations due to PWS, we were also able to exclude participants from whom only one valid examination was available or who were lost to follow-up. To the best of our knowledge, there are no prior studies in other paediatric study populations that directly compare longitudinal individual PSG evaluations before and after a COVID-19 infection.

Although on 4th May 2023 the World Health Organization declared COVID-19 to be an ongoing and established health issue which no longer constitutes a public health emergency of international concern [47], our study shows that we should continue to conduct studies on COVID-19 infection in PWS, in order to learn more about possible long-term consequences and possible treatment options to ensure optimal patient care.

## Conclusion

In our study cohort, PWS patients experienced only mild to medium symptoms during a COVID-19 infection and no participant was hospitalised. Nevertheless, after an average of three months following COVID-19 infection, differences in PSG were still apparent when compared to pre-Covid-19 examinations, manifesting in lower oxygen saturations and more frequent hypopnoea. In comparison, we observed no differences in two consecutive examinations in our control group. It remains unclear what mechanism caused this deterioration in the COVID-19 group and how long it will last. More studies are needed to evaluate possible long-term consequences and the resulting clinical implications in treatment and frequency of follow-up PSG evaluations for PWS patients. Long-term sequalae in non-PWS patients should be considered.

## Data Availability

The data that support the findings of this study are not openly available due to reasons of sensitivity and are available from the corresponding author upon reasonable request. Data are located in controlled access storage at Children`s Hospital, University of Bonn.

## Abbreviations

PWS: Prader-Willi syndrome
COVID-19: coronavirus disease 2019
PSG: overnight polysomnography
OSA: obstructive sleep apnoea
SpO2: oxygen saturation measured by pulse oximetry
CPAP: continuous positive air pressure
ODI: oxygen desaturation index
SDS_PWS_: standard deviation scores for children with Prader-Willi syndrome
SDS_healthy_: standard deviation score for healthy German children
BMI: body mass index
UPD: uniparental disomy
SD: standard deviations
Δs: delta values (post COVID-19 – pre COVID-19)

## References

[1] Butler MG, Miller JL, Forster JL. Prader-Willi Syndrome - Clinical Genetics, diagnosis and treatment approaches: an update. Curr Pediatr Rev. 2019;15:207–44. 10.2174/1573396315666190716120925. 31333129 10.2174/1573396315666190716120925 PMC7040524

[2] Butler MG. Prader-Willi Syndrome: obesity due to genomic imprinting. Curr Genomics. 2011;12:204–15. 10.2174/138920211795677877. 22043168 10.2174/138920211795677877 PMC3137005

[3] Crinò A, Fintini D, Bocchini S, Grugni G. Obesity management in Prader-Willi syndrome: current perspectives. Diabetes Metab Syndr Obes. 2018;11:579–93. 10.2147/DMSO.S141352. 30323638 10.2147/DMSO.S141352 PMC6175547

[4] Gumus Balikcioglu P, Balikcioglu M, Muehlbauer MJ, Purnell JQ, Broadhurst D, Freemark M, Haqq AM. Macronutrient Regulation of Ghrelin and peptide YY in Pediatric obesity and prader-Willi Syndrome. J Clin Endocrinol Metab. 2015;100:3822–31. 10.1210/jc.2015-2503. 26259133 10.1210/jc.2015-2503 PMC5399503

[5] Johnson L, Manzardo AM, Miller JL, Driscoll DJ, Butler MG. Elevated plasma oxytocin levels in children with prader-Willi syndrome compared with healthy unrelated siblings. Am J Med Genet A. 2016;170:594–601. 10.1002/ajmg.a.37488. 26615966 10.1002/ajmg.a.37488 PMC6679917

[6] Deal CL, Tony M, Höybye C, Allen DB, Tauber M, Christiansen JS. GrowthHormone Research Society workshop summary: consensus guidelines for recombinant human growth hormone therapy in Prader-Willi syndrome. J Clin Endocrinol Metab. 2013;98:E1072–87. 10.1210/jc.2012-3888. 23543664 10.1210/jc.2012-3888 PMC3789886

[7] Ingram DG, Arganbright JM, Paprocki E, Halpin KL. Sleep disorders in children with Prader Willi Syndrome: current perspectives. Nat Sci Sleep. 2022;14:2065–74. 10.2147/NSS.S361518. 36394064 10.2147/NSS.S361518 PMC9662031

[8] Itani R, Gillett ES, Perez IA. Sleep consequences of Prader-Willi Syndrome. Curr Neurol Neurosci Rep. 2023;23:25–32. 10.1007/s11910-023-01254-6. 36790642 10.1007/s11910-023-01254-6 PMC10011275

[9] Cucinotta D, Vanelli M. WHO declares COVID-19 a pandemic. Acta Biomed. 2020;91:157–60. 10.23750/abm.v91i1.9397. 32191675 10.23750/abm.v91i1.9397 PMC7569573

[10] Cui X, Zhao Z, Zhang T, Guo W, Guo W, Zheng J, et al. A systematic review and meta-analysis of children with coronavirus disease 2019 (COVID-19). J Med Virol. 2021;93:1057–69. 10.1002/jmv.26398. 32761898 10.1002/jmv.26398 PMC7436402

[11] Hu B, Guo H, Zhou P, Shi Z-L. Characteristics of SARS-CoV-2 and COVID-19. Nat Rev Microbiol. 2021;19:141–54. 10.1038/s41579-020-00459-7. 33024307 10.1038/s41579-020-00459-7 PMC7537588

[12] Zhang J-J, Dong X, Liu G-H, Gao Y-D. Risk and protective factors for COVID-19 morbidity, severity, and Mortality. Clin Rev Allergy Immunol. 2023;64:90–107. 10.1007/s12016-022-08921-5. 35044620 10.1007/s12016-022-08921-5 PMC8767775

[13] Maas MB, Kim M, Malkani RG, Abbott SM, Zee PC. Obstructive sleep apnea and risk of COVID-19 infection, hospitalization and respiratory failure. Sleep Breath. 2021;25:1155–7. 10.1007/s11325-020-02203-0. 32989673 10.1007/s11325-020-02203-0 PMC7521948

[14] Antoon JW, Grijalva CG, Thurm C, Richardson T, Spaulding AB, Teufel RJ, et al. Factors Associated with COVID-19 Disease Severity in US children and adolescents. J Hosp Med. 2021;16:603–10. 10.12788/jhm.3689. 34613896 10.12788/jhm.3689 PMC8494279

[15] Coupaye M, Laurier V, Benvegnu G, Poitou C, Faucher P, Mosbah H, et al. Paradoxical low severity of COVID-19 in Prader-Willi syndrome: data from a French survey on 647 patients. Orphanet J Rare Dis. 2021;16:325. 10.1186/s13023-021-01949-4. 34289876 10.1186/s13023-021-01949-4 PMC8294211

[16] Whittington JE, Holland AJ, Driscoll DJ, Hodebeck-Stuntebeck N, Hoctor A. The presentation, course, and outcome of COVID-19 infection in people with Prader-Willi syndrome: unexpected findings from an international survey. Orphanet J Rare Dis. 2022;17:69. 10.1186/s13023-022-02228-6. 35189933 10.1186/s13023-022-02228-6 PMC8860280

[17] Mohr AK, Laemmer C, Schulte S, Gohlke B. Effects of COVID-19 Lockdown on Weight, body composition, and behavior of children, adolescents, and young adults with prader-Willi Syndrome. J Clin Med. 2021. 10.3390/jcm10204746. 34682869 10.3390/jcm10204746 PMC8541437

[18] Sharma A, Metzger D. :AnthroCalc app for Android and iOS: Z-scores for children with a number of syndroms. Prader–Willi, Russell–Silver and Noonan: Turner, Down; 2022.

[19] Kromeyer-Hauschild K, Wabitsch M, Kunze D, Geller F, Geiß HC, Hesse V, et al. Perzentile für den body-mass-index für das Kindes- Und Jugendalter Unter Heranziehung verschiedener deutscher Stichproben. Monatsschr Kinderheilkd. 2001;149:807–18. 10.1007/s001120170107.

[20] Babyak MA. What you see may not be what you get: a brief, nontechnical introduction to overfitting in regression-type models. Psychosom Med. 2004;66:411–21. 10.1097/01.psy.0000127692.23278.a9. 15184705 10.1097/01.psy.0000127692.23278.a9

[21] Rothman KJ. Epidemiology: an introduction. Oxford: Oxford University Press; 2012.

[22] Butler MG, Lee PDK, Whitman BY, editors. Management of Prader-Willi Syndrome. 3rd ed. New York, NY: Prader-Willi Syndrome Association (USA); 2006.

[23] Sobolewska-Pilarczyk M, Pokorska-Śpiewak M, Stachowiak A, Marczyńska M, Talarek E, Ołdakowska A, et al. COVID-19 infections in infants. Sci Rep. 2022;12:7765. 10.1038/s41598-022-11068-0. 35546159 10.1038/s41598-022-11068-0 PMC9094122

[24] Rahman A, Tabassum T, Araf Y, Al Nahid A, Ullah MA, Hosen MJ. Silent hypoxia in COVID-19: pathomechanism and possible management strategy. Mol Biol Rep. 2021;48:3863–9. 10.1007/s11033-021-06358-1. 33891272 10.1007/s11033-021-06358-1 PMC8062941

[25] Wu X, Liu X, Zhou Y, Yu H, Li R, Zhan Q, et al. 3-month, 6-month, 9-month, and 12-month respiratory outcomes in patients following COVID-19-related hospitalisation: a prospective study. Lancet Respir Med. 2021;9:747–54. 10.1016/S2213-2600(21)00174-0. 33964245 10.1016/S2213-2600(21)00174-0 PMC8099316

[26] Dhont S, Derom E, van Braeckel E, Depuydt P, Lambrecht BN. The pathophysiology of ‘happy’ hypoxemia in COVID-19. Respir Res. 2020;21:198. 10.1186/s12931-020-01462-5. 32723327 10.1186/s12931-020-01462-5 PMC7385717

[27] Bepouka B, Odio O, Mayasi N, Longokolo M, Mangala D, Mandina M, et al. Prevalence and outcomes of COVID – 19 patients with happy hypoxia: a systematic review. Infect Drug Resist. 2022;15:5619–28. 10.2147/IDR.S378060. 36172621 10.2147/IDR.S378060 PMC9512283

[28] Okuhama A, Ishikane M, Hotta M, Sato L, Akiyama Y, Morioka S, et al. Clinical and radiological findings of silent hypoxia among COVID-19 patients. J Infect Chemother. 2021;27:1536–8. 10.1016/j.jiac.2021.07.002. 34294527 10.1016/j.jiac.2021.07.002 PMC8264520

[29] Fuglebjerg NJU, Jensen TO, Hoyer N, Ryrsø CK, Lindegaard B, Harboe ZB. Silent hypoxia in patients with SARS CoV-2 infection before hospital discharge. Int J Infect Dis. 2020;99:100–1. 10.1016/j.ijid.2020.07.014. 32663601 10.1016/j.ijid.2020.07.014 PMC7836996

[30] Elmer N, Liebl ME, Schwedtke C, Drebinger D, Reißhauer A. Akutrehabilitation Nach COVID–19–Infektion: Eine ausgewählte Fallserie. [Acute rehabilitation after COVID-19 infection: a selected case series]. Z Rheumatol. 2022;81:386–92. 10.1007/s00393-022-01178-5. 35318531 10.1007/s00393-022-01178-5 PMC8939397

[31] Gozal D, Arens R, Omlin KJ, Ward SL, Keens TG. Absent peripheral chemosensitivity in Prader-Willi syndrome. J Appl Physiol (1985). 1994;77:2231–6. 10.1152/jappl.1994.77.5.2231. 7868439 10.1152/jappl.1994.77.5.2231

[32] Arens R, Gozal D, Omlin KJ, Livingston FR, Liu J, Keens TG, Ward SL. Hypoxic and hypercapnic ventilatory responses in Prader-Willi syndrome. J Appl Physiol (1985). 1994;77:2224–30. 10.1152/jappl.1994.77.5.2224. 7868438 10.1152/jappl.1994.77.5.2224

[33] Arens R, Gozal D, Burrell BC, Bailey SL, Bautista DB, Keens TG, Ward SL. Arousal and cardiorespiratory responses to hypoxia in Prader-Willi syndrome. Am J Respir Crit Care Med. 1996;153:283–7. 10.1164/ajrccm.153.1.8542130. 8542130 10.1164/ajrccm.153.1.8542130

[34] Lecka-Ambroziak A, Wysocka-Mincewicz M, Świercz A, Jędrzejczak M, Szalecki M. Comparison of frequency and severity of Sleep-Related Breathing disorders in children with Simple Obesity and paediatric patients with prader-Willi Syndrome. J Pers Med. 2021. 10.3390/jpm11020141. 33670584 10.3390/jpm11020141 PMC7923084

[35] Fidan V, Koyuncu H, Akin O. Alteration of Auto-CPAP requirements in obstructive sleep apnea patients with COVID-19 history. Am J Otolaryngol. 2021;42:102919. 10.1016/j.amjoto.2021.102919. 33476971 10.1016/j.amjoto.2021.102919 PMC7832370

[36] Mogensen I, Hallberg J, Björkander S, Du L, Zuo F, Hammarström L, et al. Lung function before and after COVID-19 in young adults: a population-based study. J Allergy Clin Immunol Glob. 2022;1:37–42. 10.1016/j.jacig.2022.03.001. 36647376 10.1016/j.jacig.2022.03.001 PMC8966371

[37] Soyak Aytekin E, Sahiner UM, Tuten Dal S, Unsal H, Hakverdi O, Oguz B, et al. Obesity is a risk factor for decrease in lung function after COVID-19 infection in children with asthma. Pediatr Pulmonol. 2022;57:1668–76. 10.1002/ppul.25949. 35502514 10.1002/ppul.25949 PMC9347415

[38] Lau EYY, Choi EWM, Lai ESK, Lau KNT, Au CT, Yung WH, Li AM. Working memory impairment and its associated sleep-related respiratory parameters in children with obstructive sleep apnea. Sleep Med. 2015;16:1109–15. 10.1016/j.sleep.2015.04.025. 26298787 10.1016/j.sleep.2015.04.025

[39] Bourke R, Anderson V, Yang JSC, Jackman AR, Killedar A, Nixon GM, et al. Cognitive and academic functions are impaired in children with all severities of sleep-disordered breathing. Sleep Med. 2011;12:489–96. 10.1016/j.sleep.2010.11.010. 21493135 10.1016/j.sleep.2010.11.010

[40] Gillett ES, Perez IA. Disorders of Sleep and Ventilatory Control in Prader-Willi Syndrome. Diseases. 2016. 10.3390/diseases4030023. 28933403 10.3390/diseases4030023 PMC5456282

[41] Turner K, Zambrelli E, Lavolpe S, Baldi C, Furia F, Canevini MP. Obstructive sleep apnea: neurocognitive and behavioral functions before and after treatment. Funct Neurol. 2019;34:71–8. 31556386

[42] Choi JH, Choi S-H, Yun KW. Risk factors for severe COVID-19 in children: a systematic review and Meta-analysis. J Korean Med Sci. 2022;37:e35. 10.3346/jkms.2022.37.e35. 35132841 10.3346/jkms.2022.37.e35 PMC8822112

[43] Ahrenfeldt LJ, Otavova M, Christensen K, Lindahl-Jacobsen R. Sex and age differences in COVID-19 mortality in Europe. Wien Klin Wochenschr. 2021;133:393–8. 10.1007/s00508-020-01793-9. 33351155 10.1007/s00508-020-01793-9 PMC7755064

[44] Raza HA, Sen P, Bhatti OA, Gupta L. Sex hormones, autoimmunity and gender disparity in COVID-19. Rheumatol Int. 2021;41:1375–86. 10.1007/s00296-021-04873-9. 33903964 10.1007/s00296-021-04873-9 PMC8075025

[45] Torrado M, Araoz V, Baialardo E, Abraldes K, Mazza C, Krochik G, et al. Clinical-etiologic correlation in children with prader-Willi syndrome (PWS): an interdisciplinary study. Am J Med Genet A. 2007;143A:460–8. 10.1002/ajmg.a.31520. 17163531 10.1002/ajmg.a.31520

[46] Cintra HA, Rocha DN, da Costa ACC, Tyszler LS, Freitas S, de Araujo LA, et al. Investigating the correlation between genotype and phenotype in Prader-Willi syndrome: a study of 45 cases from Brazil. Orphanet J Rare Dis. 2024;19:240. 10.1186/s13023-024-03157-2. 38902749 10.1186/s13023-024-03157-2 PMC11188222

[47] World Health Organization. Statement on the fifteenth meeting of the IHR. (2005) Emergency Committee on the COVID-19 pandemic. 5 May 2023. https://www.who.int/news/item/05-05-2023-statement-on-the-fifteenth-meeting-of-the-international-health-regulations-(2005)-emergency-committee-regarding-the-coronavirus-disease-(covid-19)-pandemic. Accessed 18 May 2023.

